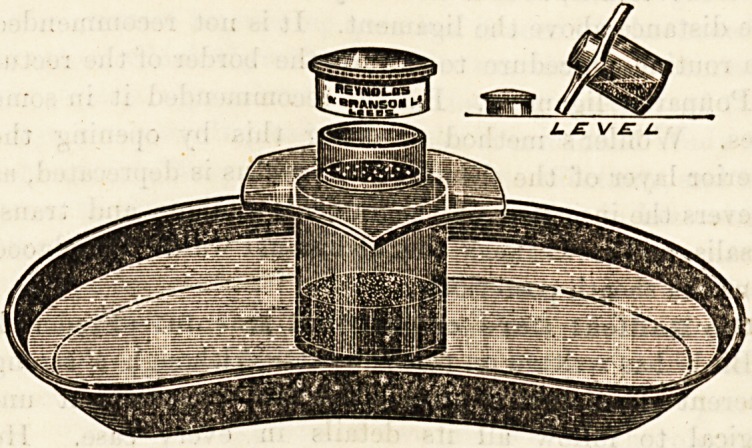# New Appliances and Things Medical

**Published:** 1900-12-08

**Authors:** 


### NEW APPLIANCES AND THINGS MEDICAL.

[We shall be glad to receive, at our Office, 28 & 29 Southampton Street, Strand, London, W.C., from the manufacturers, specimens of all new preparations and appliances which may be brought out from time to time.]

### TROPELS AND PASTILLES.

(Three Spires Brand, Wyley's, Limited, Coventry.)

We have examined a number of samples of Tropels and Pastilles forwarded us from Wyley's, Limited, also Neuracetin Pellets. Among the tropels we would especially call attention to the digestive, cough, voice, and bronchial varieties. Although it is impossible here to detail the formulæ on which their preparation is based, we may remark that they are composed of drugs which deservedly hold a high position in medical estimation. Among the Pastilles may be mentioned menthol and eucalyptus, boric acid and antiseptic throat varieties. The active medicaments contained in these preparations are in safe and useful proportion: they are as pleasant to the taste as the nature of the constituents will allow, and should be invaluable in maintaining the throat and fauces in a healthy condition. Pastilles or Tropels can be prepared in any desired formula, and practitioners will do well to avail themselves of the artistic methods of Messrs. Wyley in supplying particular combinations of drugs for the benefit of their patients. Neuracetin possesses the combined properties of an antipyretic, analgesic, antineuralgic, and anodyne, and being similar but not identical with several other popular drugs of the same nature, it will be found a valuable substitute for cases in which, from long familiarity, these drugs have become less efficacious.

### "GEM" PURE WATER STILL.

(The Gem Supplies Company, 6 Bishop's Court, Chancery Lane, London, W.C.)

There are cases in which it is desirable to use distilled water as a beverage, either alone or as a basis for the

A. The Retort. Where raw water is generated into steam.
B. Condenser Reservoir. Contains cold water.
C. Condensing Chamber. Where steam is condensed.
D. Distillate Reservoir. Where distilled water is stored ready for use.
E. Sterilizing Chamber. For purifying the air used.
F. Feed Cup and Indicator. Used to replenish A. Shows the amount of water in Retort.
G. Ventilating Flue.
H. Distillate Faucet. For drawing off distilled water.
I. Condenser Faucet. Used to fill retort A.
J. Distillate Overflow. Prevents running over or boiling dry.

making of drinks. Sometimes it happens that the only available water supply is known to be contaminated, much more frequently that its purity is doubtful, and in either case it becomes a serious question how to obtain a safe supply. Among the several methods available there can hardly be a doubt that from the standpoint of purity distillation is the most perfect means at our disposal. There have, however, been many difficulties in the way of domestic distillation, partly from the complexity of the apparatus and partly from the lack of a place in which to put it in an ordinary house. The "Gem" Pure Water Still, however, makes all very simple. First of all the apparatus can stand on the hot plate of the stove, and occupies no more space than a Warren's cooker or a potato steamer; next, the economy of heat is very considerable, the latent heat in the steam being continually imparted, in the act of condensation, to the water which is about to go into the boiler. There is thus but little of that waste of heat which is so marked a feature of some of the older types of stills. Lastly, it requires very little more attention than a potato steamer. The appended diagrams will explain the action of the apparatus, which is simplicity itself.

### AN ASEPTIC DREDGER.

(Reynolds and Branson, Limited, Briggate, Leeds.)

This new form of dredger is designed for the purpose of rendering more complete the methods of aseptic surgery. As will be seen from the illustration, it is provided with a wide flange or rim which in the first place keeps the dredger in an upright position when floating in an antiseptic solution, and secondly protects the object which is being dusted with the contained powder from any material, whether solid or fluid, which may drop from the surgeon's hands when completing the dressing. The dredger is well designed for keeping the contained powder free from contamination, and for floating in an antiseptic lotion during the stages of an operation. It appears, however, rather an unnecessary refinement of asepticity.

## Figures and Tables

**Figure f1:**
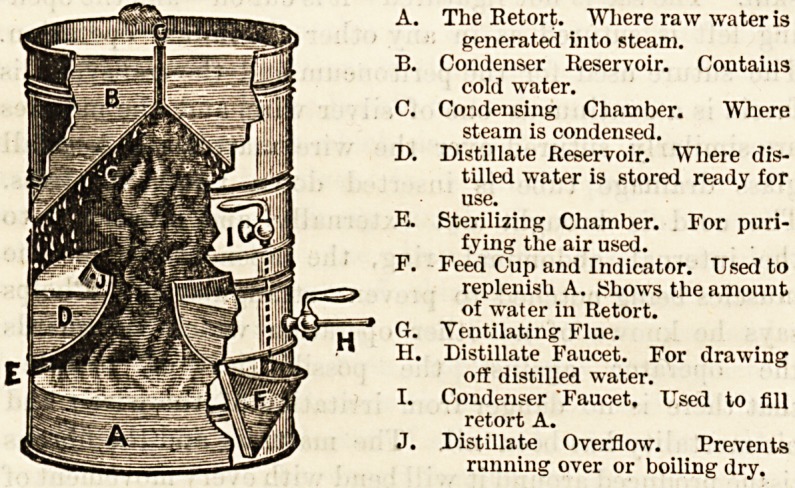


**Figure f2:**